# Validity of U.S. Nutritional Surveillance: National Health and Nutrition Examination Survey Caloric Energy Intake Data, 1971–2010

**DOI:** 10.1371/journal.pone.0076632

**Published:** 2013-10-09

**Authors:** Edward Archer, Gregory A. Hand, Steven N. Blair

**Affiliations:** 1 Department of Exercise Science, Arnold School of Public Health, University of South Carolina, Columbia, South Carolina, United States of America; 2 Department of Epidemiology and Biostatistics, University of South Carolina, Columbia, South Carolina, United States of America; Pennington Biomed Research Center, United States of America

## Abstract

**Importance:**

Methodological limitations compromise the validity of U.S. nutritional surveillance data and the empirical foundation for formulating dietary guidelines and public health policies.

**Objectives:**

Evaluate the validity of the National Health and Nutrition Examination Survey (NHANES) caloric intake data throughout its history, and examine trends in the validity of caloric intake estimates as the NHANES dietary measurement protocols evolved.

**Design:**

Validity of data from 28,993 men and 34,369 women, aged 20 to 74 years from NHANES I (1971–1974) through NHANES 2009–2010 was assessed by: calculating physiologically credible energy intake values as the ratio of reported energy intake (rEI) to estimated basal metabolic rate (BMR), and subtracting estimated total energy expenditure (TEE) from NHANES rEI to create ‘disparity values’.

**Main Outcome Measures:**

1) Physiologically credible values expressed as the ratio rEI/BMR and 2) disparity values (rEI–TEE).

**Results:**

The historical rEI/BMR values for men and women were 1.31 and 1.19, (95% CI: 1.30–1.32 and 1.18–1.20), respectively. The historical disparity values for men and women were −281 and −365 kilocalorie-per-day, (95% CI: −299, −264 and −378, −351), respectively. These results are indicative of significant under-reporting. The greatest mean disparity values were −716 kcal/day and −856 kcal/day for obese (i.e., ≥30 kg/m2) men and women, respectively.

**Conclusions:**

Across the 39-year history of the NHANES, EI data on the majority of respondents (67.3% of women and 58.7% of men) were not physiologically plausible. Improvements in measurement protocols after NHANES II led to small decreases in underreporting, artifactual increases in rEI, but only trivial increases in validity in subsequent surveys. The confluence of these results and other methodological limitations suggest that the ability to estimate population trends in caloric intake and generate empirically supported public policy relevant to diet-health relationships from U.S. nutritional surveillance is extremely limited.

## Introduction

The rise in the population prevalence of obesity has focused attention on U.S. nutritional surveillance research and the analysis of trends in caloric energy intake (EI). Because these efforts provide the scientific foundation for many public health policies and food-based guidelines, poor validity in dietary measurement protocols can have significant long-term implications for our nation’s health.

In the U.S., population-level estimates of EI are derived from data collected as part of the National Health and Nutrition Examination Survey (NHANES), a complex, cross-sectional sample of the U.S. population. The primary method used in NHANES to approximate EI is the 24-hour dietary recall interview (24HR) [1]. The data collected are based on the subject’s self-reported, retrospective perceptions of food and beverage consumption in the recent past. To calculate EI estimates, these subjective data are translated into nutrient food codes and then assigned numeric energy (i.e., caloric) values from food and nutrient databases. Prior to 2001–2002, the NHANES relied upon databases of varying quality and composition for the *post-hoc* conversion of food and beverage consumption (24HR) data into energy values [2]–[5]. After 2001–2002, the NHANES and the U.S. Department of Agriculture’s (USDA) Continuing Survey of Food Intakes by Individuals were integrated into the “What We Eat in America” program [6], and the translation process was standardized via use of successive versions of the USDA’s National Nutrient Database for Standard Reference (NNBS) [7].

### Misreporting

Given the indirect, pseudo-quantitative nature of the method (i.e., assigning numeric values to subjective data without objective corroboration), nutrition surveys frequently report a range of energy intakes that are not representative of the respondents’ habitual intakes [8], and estimates of EI that are physiologically implausible (i.e., incompatible with survival) have been demonstrated to be widespread [9]–[11]. For example, in a group of “highly educated” participants, Subar et al. (2003) demonstrated that when total energy expenditure (TEE) via doubly labeled water (DLW) was compared to reported energy intake (rEI), the raw correlations between TEE and rEI were 0.39 for men and 0.24 for women. Men and women underreported energy intake by 12–14% and 16–20%, respectively. The level of underreporting increased significantly after correcting for the weight gain of the sample over the study period [9], and underreporting was greater for fat than for protein, thereby providing additional support for the well-documented occurrence of the selective misreporting of specific macronutrients (e.g., fat and sugars) [12]–[15]. These results are consistent with earlier work, in which the correlations between DLW-derived TEE and seven 24HR and the average of two seven-day dietary recalls were 0.33 and 0.30, respectively [16].

Because the NHANES collected dietary data over the period in which the population prevalence of obesity was increasing, these data have been used (despite the widely acknowledged issues [17]) to examine the association of trends in EI with increments in mean population body mass index (BMI) and rates of obesity (e.g., [18]–[20]). Given that implausible rEI values and the misreporting of total dietary intake render the relationships between dietary factors, BMI and other indices of health ambiguous [21], and diminish the usefulness of nutrition data as a tool to inform public health policy, this report examines the validity of U.S. nutrition surveillance EI data from NHANES I (1971–1974) through NHANES 2010 (nine survey periods) using two protocols: the ratio of reported energy intake (rEI) to basal metabolic rate (rEI/BMR) [22], [23] and the disparity between rEI and estimated total energy expenditure (TEE) from the Institute of Medicine’s (IOM) predictive equations [24].

## Methods

### Population

Data were obtained from the National Health and Nutrition Examination Surveys for the years 1971–2010 [1]. The NHANES is a complex multi-stage, cluster sample of the civilian, non-institutionalized U.S. population conducted by the Centers for Disease Control and Prevention (CDC). The National Center for Health Statistics ethics review board approved protocols and written informed consent was obtained from all NHANES participants.

### Inclusion Criteria

The study sample was limited to adults aged ≥20 and <74 years at the time of the NHANES in which they participated, and had a body mass index (BMI) ≥18 kg/m^2^, and with complete data on age, sex, height, weight, and dietary energy intake.

### Dietary Data

Estimates of EI were obtained from a single 24HR from each of the nine NHANES study periods [1]. Energy content of the self-reported food consumption was determined by NHANES using nutrient databases based on previous versions of the USDA National Nutrient Database for Standard Reference (NNDS) [7].

### Determination of Physiologically Credible rEI Values

The ratio of rEI to BMR (rEI/BMR) <1.35 [22], [23], [25] was used to determine EI values that were implausible. BMR was estimated via the Schofield predictive equations [26]. The <1.35 cut-off for implausible EI values was used because “*it is highly unlikely that any normal, healthy free-living person could habitually exist at a PAL* [i.e., TEE/BMR] *of less than 1.35”* [22].

It is important to note that the <1.35 cut-off does not assess all forms of misreporting (e.g., over-reporting). To avoid the confounding effects of potential over-reporting, all rEI/BMR values >2.40 [27] were excluded from analyses of underreporting. One form of misreporting that neither cut-off addresses is the underreporting of EI from a high caloric intake associated with elevated levels of physical activity.

### Disparity of the rEI and Estimated Total Energy Expenditure (TEE)

In 2002, the IOM used datasets derived from studies using DLW to create factorial equations to estimate energy requirements for the US population. IOM TEE values were subtracted from the NHANES rEI to calculate disparity values. Negative values indicate underreporting.

### IOM Equations for Predicting TEE Normal Weight (NW) Adults only (≥19years)

Equation 1 Men: TEE = 864– (9.72×age [y])+PA*×(14.2×weight [kg]+503×height[m]) (±202).

Equation 2 Women: TEE = 387– (7.31×age [y]+PA*×(10.8×weight [kg]+660.7×height[m]) (±156).

* Physical activity (PA) values were 1.12 and 1.14 for NW men and women, respectively. The use of these values assumes a physical activity level (PAL) of ≥1.4 and <1.6, which is indicative of a “low active” population [24].

### IOM Equations for Predicting TEE Overweight (OW)/Obese (OB) Adults Only (≥19 years)

Equation 3 Men: TEE = 1086– (10.1×age [y])+PA*×(13.7×weight [kg]+416×height [m]).

Equation 4 Women: TEE = 448– (7.95×age [y])+PA*×(11.4×weight [kg]+619×height [m]).

*PA values were 1.12 and 1.16 for OW/OB men and women, respectively. The use of these values assumes a physical activity level (PAL) of ≥1.4 and <1.6, which is indicative of a “low active” population [24].

Note: age (years); weight (kg); height (m; meters); BMI = body mass index, (kg/m^2^), IOM = Institute of Medicine; TEE = total energy expenditure.

### Anthropometry [1]

Body mass was measured to ±0.1 kg. Height was measured to ±0.1 cm. BMI was calculated as weight (kg)/height (m)^2^. The sample was divided into three standard BMI categories: BMI ≥18 kg/m^2^ and <25 kg/m^2^ were normal weight (NW), BMI between 25 kg/m^2^ and 29.9 kg/m^2^ were overweight (OW), and ≥30 kg/m^2^ were obese (OB).

### Statistical Analyses

Data processing and statistical analyses were performed using SAS®, V 9.2 and SPSS® V.19 in 2012–2013. Analyses accounted for the NHANES’ complex survey design via the incorporation of stratification, clustering and post-stratification weighting to maintain a nationally representative sample for each survey period. All analyses included adjusted means, and *α* <0.05 (2-tailed) was used to identify statistical significance.

## Results

### Examination of Underreporting via rEI/BMR

Table 1 depicts the rEI/BMR values for men and women from NHANES I through NHANES 2009–2010. rEI was from NHANES 24HR data and BMR was calculated using the Schofield predictive equations [26]. Values <1.35 are considered implausible and indicative of underreporting.

**Table 1:** rEI/BMR values for all men and women from NHANES I through NHANES 2009–2010.

Reported Energy Intake (rEI)/Basal Metabolic Rate (BMR) rEI/BMR >1.35 = plausible US Men & Women (20–74 years); NHANES I - NHANES 2009–2010						
NHANESSurvey Year	Sex	Estimate rEI/RMR (mean)*	Standard Error	95% Confidence Interval		rEI Value Plausible Y = Yes N = No
				Lower	Upper	
NHANES I	Men (n = 4652)	1.30	0.012	1.28	1.32	N
	Women (n = 7709)	1.10	0.010	1.08	1.12	N
NHANES II	Men (n = 5236)	1.28	0.010	1.26	1.30	N
	Women (n = 6006)	1.08	0.008	1.06	1.09	N
NHANES III	Men (n = 6122)	1.36^b^	0.011	1.34	1.39	Y
	Women (n = 7127)	1.22^a^	0.009	1.20	1.24	N
NHANES I999–00	Men (n = 1600)	1.31	0.018	1.27	1.34	N
	Women (n = 1886)	1.23^a^	0.016	1.19	1.26	N
NHANES 2001–2002	Men (n = 1782)	1.31	0.015	1.28	1.34	N
	Women (n = 2029)	1.24^a^	0.011	1.22	1.26	N
NHANES 2003–2004	Men (n = 1671)	1.32	0.013	1.30	1.35	Y
	Women (n = 1838)	1.23^a^	0.018	1.20	1.27	N
NHANES 2005–2006	Men (n = 1749)	1.34^c^	0.013	1.31	1.36	Y
	Women (n = 1998)	1.21^a^	0.014	1.18	1.24	N
NHANES 2007–08	Men (n = 2154)	1.27	0.017	1.24	1.30	N
	Women (n = 2306)	1.19^a^	0.020	1.15	1.23	N
NHANES 2009–2010	Men (n = 2319)	1.29	0.013	1.26	1.31	N
	Women (n = 2532)	1.20^a^	0.007	1.18	1.21	N
**All Surveys**	**Men (n = 27285)**	**1.31**	**0.005**	**1.30**	**1.32**	**N**
	**Women (n = 33431)**	**1.19**	**0.005**	**1.18**	**1.20**	**N**

* All estimates are weighted means.

a Significantly different from NHANES I at p≤0.001 (Women).

b Significantly different from NHANES I at p≤0.001 (Men).

c Significantly different from NHANES I at p≤0.05 (Men).

Note: rEI was from NHANES 24HR data and BMR was calculated using the Schofield predictive equations. [26] Values <1.35 are considered implausible and indicative of underreporting. TEE = estimated total energy expenditure; IOM = Institute of Medicine; rEI = reported energy intake; BMR = Basal Metabolic Rate calculated via Schofield predictive equation.

Values <1.35 are not physiologically credible.

As Table 1 depicts, the 95% confidence intervals (CI) suggest that all mean rEI values for women and six of nine mean rEI values for men were apparently implausible.

Table 2 depicts the rEI/BMR index for all women by BMI categories from NHANES I through NHANES 2009–2010.

**Table 2:** rEI/BMR index for all women by BMI categories from NHANES I through NHANES 2009–2010.

Reported Energy Intake (rEI)/Basal Metabolic Rate (BMR) rEI/BMR >1.35 = plausible US Women (20–74 years); NHANES I - NHANES 2009–2010						
NHANESSurvey Year	BMI Category	EstimaterEI/BMR(Mean)*	Standard Error	95% Confidence Interval		rEI Value Plausible Y = Yes N = No
				Lower	Upper	
NHANES I	Normal (n = 4222)	1.20	0.013	1.18	1.23	N
	Overweight (n = 2028)	1.00	0.012	0.98	1.02	N
	Obese (n = 1459)	0.88	0.014	0.86	0.91	N
NHANES II	Normal (n = 3171)	1.18	0.010	1.16	1.20	N
	Overweight (n = 1671)	0.98	0.012	0.96	1.01	N
	Obese (n = 1164)	0.89	0.012	0.87	0.91	N
NHANES III	Normal (n = 2661)	1.32	0.014	1.30	1.35	Y
	Overweight (n = 2150)	1.18	0.019	1.14	1.22	N
	Obese (n = 2316)	1.07	0.015	1.04	1.10	N
NHANES 1999–2000	Normal (n = 555)	1.36	0.020	1.32	1.40	Y
	Overweight (n = 572)	1.19	0.033	1.12	1.25	N
	Obese (n = 759)	1.12	0.030	1.06	1.18	N
NHANES 2001–2002	Normal (n = 630)	1.38	0.018	1.35	1.42	Y
	Overweight (n = 639)	1.26	0.028	1.21	1.32	N
	Obese (n = 760)	1.08	0.012	1.05	1.10	N
NHANES 2003–2004	Normal (n = 550)	1.35	0.031	1.29	1.41	Y
	Overweight (n = 546)	1.19	0.027	1.14	1.25	N
	Obese (n = 742)	1.15	0.026	1.10	1.20	N
NHANES 2005–2006	Normal (n = 615)	1.34	0.026	1.29	1.39	Y
	Overweight (n = 558)	1.19	0.028	1.13	1.24	N
	Obese (n = 825)	1.10	0.024	1.05	1.15	N
NHANES 2007–2008	Normal (n = 634)	1.30	0.038	1.23	1.38	Y
	Overweight (n = 694)	1.17	0.026	1.12	1.22	N
	Obese (n = 978)	1.10	0.020	1.06	1.14	N
NHANES 2009–2010	Normal (n = 690)	1.31	0.022	1.26	1.35	Y
	Overweight (n = 745)	1.23	0.024	1.18	1.28	N
	Obese (n = 1097)	1.08	0.006	1.06	1.09	N

* All estimates are weighted means.

Note: rEI was from NHANES 24HR data and BMR was calculated using the Schofield predictive equations. [26] Values <1.35 are considered implausible and indicative of underreporting. TEE = estimated total energy expenditure; IOM = Institute of Medicine; rEI = reported energy intake; BMR = Basal Metabolic Rate calculated via Schofield predictive equation.

As Table 2 depicts, the 95% CI suggest that in 20 of the 27 measurement categories (i.e., three BMI categories and nine surveys) the rEI values were not in the physiologically plausible range. The overall mean for rEI/BMR values for the total sample of women (n = 33,431) across all NHANES was 1.19 (95% CI: 1.18, 1.20) and therefore not physiologically plausible.

Table 3 depicts the rEI/BMR index for all men by BMI categories from NHANES I through NHANES 2009–2010.

**Table 3:** rEI/BMR index for all men by BMI categories from NHANES I through NHANES 2009–2010.

Reported Energy Intake (rEI)/Basal Metabolic Rate (BMR) rEI/BMR >1.35 = plausible US Men (20–74 years); NHANES I - NHANES 2009–2010						
NHANESSurvey Year	BMI Category	Estimate rEI/BMR (Mean)*	Standard Error	95% Confidence Interval		rEI Value Plausible Y = Yes N = No
				Lower	Upper	
NHANES I	Normal (n = 2115)	1.41	0.016	1.38	1.44	Y
	Overweight (n = 1945)	1.24	0.017	1.21	1.28	N
	Obese (n = 592)	1.08	0.025	1.04	1.13	N
NHANES II	Normal (n = 2431)	1.37	0.009	1.35	1.39	Y
	Overweight (n = 2111)	1.25	0.015	1.22	1.28	N
	Obese (n = 694)	1.08	0.018	1.05	1.12	N
NHANES III	Normal (n = 2275)	1.47	0.018	1.43	1.50	Y
	Overweight (n = 2482)	1.35	0.015	1.32	1.38	Y
	Obese (n = 1365)	1.20	0.018	1.17	1.24	N
NHANES 1999–2000	Normal (n = 476 )	1.42	0.020	1.38	1.46	Y
	Overweight (n = 655)	1.33	0.022	1.28	1.37	Y
	Obese (n = 469)	1.16	0.036	1.09	1.23	N
NHANES 2001–2002	Normal (n = 493)	1.43	0.038	1.35	1.50	Y
	Overweight (n = 774)	1.32	0.017	1.29	1.36	Y
	Obese (n = 515)	1.18	0.027	1.13	1.24	N
NHANES 2003–2004	Normal (n = 465)	1.46	0.029	1.41	1.52	Y
	Overweight (n = 659)	1.35	0.025	1.30	1.40	Y
	Obese (n = 547)	1.18	0.035	1.11	1.24	N
NHANES 2005–2006	Normal (n = 413)	1.51	0.030	1.45	1.57	Y
	Overweight (n = 735)	1.33	0.023	1.29	1.38	Y
	Obese (n = 601)	1.22	0.014	1.19	1.25	N
NHANES 2007–2008	Normal (n = 539)	1.40	0.038	1.32	1.47	Y
	Overweight (n = 835)	1.29	0.017	1.26	1.32	N
	Obese (n = 790)	1.15	0.019	1.12	1.19	N
NHANES 2009–2010	Normal (n = 563)	1.38	0.027	1.33	1.44	Y
	Overweight (n = 872)	1.35	0.021	1.31	1.39	Y
	Obese (n = 884)	1.16	0.016	1.13	1.19	N

* All estimates are weighted means.

Note: rEI was from NHANES 24HR data and BMR was calculated using the Schofield predictive equations. [26] Values <1.35 are considered implausible and indicative of underreporting. TEE = estimated total energy expenditure; IOM = Institute of Medicine; rEI = reported energy intake; BMR = Basal Metabolic Rate calculated via Schofield predictive equation.

As shown in Table 3, the 95% CI suggest that in 12 of 27 measurement categories (i.e., three BMI categories and nine surveys), the rEI values were not in the physiologically plausible range. The overall mean value for rEI/BMR for the total sample of men (n = 27,285) across all NHANES was 1.31 (95% CI: 1.30, 1.32), and therefore not in the physiologically plausible range.

### Percent of Plausible Reporters

**Figure 1:**
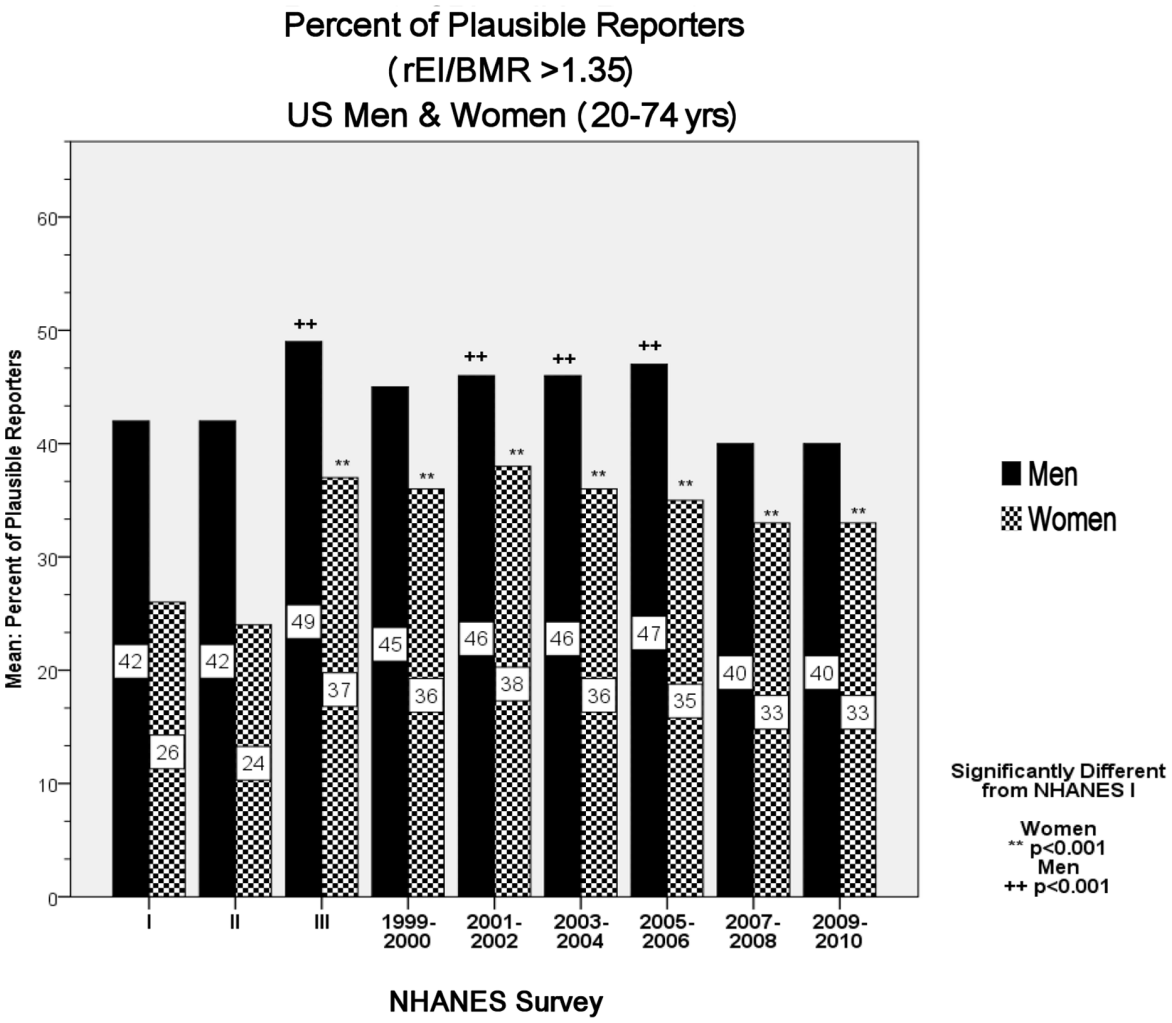
Percent of plausible reporters (i.e., rEI/BMR >1.35) by sex from NHANES I to NHANES 2009–2010; U.S. Men and women (20–74 years).

Figure 1 depicts the percent of plausible reporters (i.e., rEI/BMR >1.35) by sex from NHANES I to NHANES 2009–2010.

As Figure 1 depicts, across the entire study period (i.e., 1971–2010) the majority of respondents did not report plausible rEI values in any survey. When stratified by sex and BMI categories, plausible reporting in OB women ranged from a low of ∼12% in NHANES I and II to a high of 31% in NHANES 2003–2004. At no point in the history of the NHANES did more than 43% of OW and OB women report plausible values. Plausible reporting in NW women ranged from a low of 32% in NHANES II to 52% in NHANES 2001–2002. Plausible rEI values in OB men ranged from a low of 23% in NHANES II to a high of 35% in NHANES 2005–2006. At no point in the history of NHANES did more than 49% of OW and OB men report plausible rEI values.

### Disparity between NHANES rEI and IOM TEE

Table 4 depicts the disparity of rEI and TEE for men and women (20–74 years). These values were calculated by subtracting the IOM TEE from the NHANES rEI. Negative values indicate the kilocalorie-per-day (kcal/day) value of underreporting.

**Table 4:** Disparity of rEI and TEE for men and women (20–74 years).

Disparity between rEI and IOM TEE US Men & Women (20–74 years) NHANES I – NHANES 2009–2010						
NHANESSurvey Year	Sex	EstimaterEI minus TEE (mean)*	Standard Error	95% Confidence Interval (CI)		Validity: 95% CI includes zero (Y = Yes, N = No)
				Lower	Upper	
NHANES I	Men (n = 4652)	−290.8	20.3	−330.7	−250.9	N
	Women (n = 7709)	−479.7	14.5	−508.1	−451.3	N
NHANES II	Men (n = 5236)	−323.2	17.8	−358.1	−288.3	N
	Women (n = 6006)	−505.8	11.6	−528.4	−483.1	N
NHANES III	Men (n = 6122)	−183.3^b^	19.1	−220.8	−145.7	N
	Women (n = 7127)	−325.3^a^	13.5	−351.7	−298.8	N
NHANES 1999–2000	Men (n = 1600)	−285.3	37.7	−359.3	−211.4	N
	Women (n = 1886)	−328.7^a^	27.3	−382.3	−275.1	N
NHANES 2001–2002	Men (n = 1782)	−270.3	26.8	−322.9	−217.7	N
	Women (n = 2029)	−306.0^a^	15.5	−336.3	−275.6	N
NHANES 2003–2004	Men (n = 1671)	−255.6	24.7	−304.0	−207.3	N
	Women (n = 1838)	−308.2^a^	27.2	−361.5	−254.8	N
NHANES 2005–2006	Men (n = 1749)	−232.2	25.3	−281.8	−182.6	N
	Women (n = 1998)	−347.5^a^	20.8	−388.4	−306.6	N
NHANES 2007–08	Men (n = 2154)	−355.0	32.1	−417.9	−292.0	N
	Women (n = 2306)	−379.4^d^	28.5	−435.3	−323.5	N
NHANES 2009–2010	Men (n = 2319)	−330.9	22.7	−375.4	−286.4	N
	Women (n = 2532)	−366.9^a^	9.8	−386.1	−347.7	N
**All Surveys**	**Men (n = 27285)**	−**281.4**	**9.1**	−**299.3**	−**263.5**	**N**
	**Women (n = 33431)**	−**364.6**	**7.0**	−**378.3**	−**351.0**	**N**

* All estimates are weighted means.

a Significantly different from NHANES I at p≤0.001 (Women).

b Significantly different from NHANES I at p≤0.001 (Men).

c Significantly different from NHANES I at p≤0.05 (Men).

d Significantly different from NHANES I at p≤0.05 (Women).

Note: TEE = estimated total energy expenditure; IOM = Institute of Medicine; rEI = reported energy intake; BMR = Basal Metabolic Rate calculated via Schofield predictive equation.

These values were calculated by subtracting the IOM TEE from the NHANES rEI. Negative values indicate the kilocalorie-per-day (kcal/day) value of underreporting.

As Table 4 depicts, in no survey group (i.e., men & women in 9 surveys) does the 95% CI for the disparity between rEI and TEE include zero. This suggests that that underreporting of EI occurred in both men and women, and across all surveys. The overall mean value for the disparity of rEI and IOM TEE for the total sample of women (n = 33,431) across all NHANES was −365 kcal/day (95% CI: −378, −351), or ∼18% of TEE, and for the total sample of men (n = 27,285) was −281 kcal/day (95% CI: −299, −264), or ∼10% of TEE.

When stratified by sex and BMI categories (see *Tables* 5 & 6), the disparities between rEI and TEE in OB women ranged from −856 kcal/day (95% CI: −902, −810), an underreporting of ∼41% of TEE, to −477 kcal/day (95% CI: −560, −394), an underreporting of 20% of TEE. The disparities between rEI and TEE in OB men ranged from −717 kcal/day (95% CI: −790, −643) in NHANES II to −464 kcal/day (95% CI: −527, −401) underreporting of 25% and 15%, respectively.

**Table 5:** Disparity between rEI and the TEE for women (20–74 years) by BMI categories.

Disparity between rEI and IOM TEE; US Women by BMI categories (20–74 years) NHANES I – NHANES 2009–2010						
NHANESSurvey Year	BMI Category	Estimate rEI minus TEE (mean)	Standard Error	95% Confidence Interval (CI)		Validity: 95% CI includes zero (Y = Yes, N = No)
				Lower	Upper	
NHANES I	Normal n = 4222)	−316.0	17.7	−350.8	−281.2	N
	Overweight (n = 2028)	−595.3	17.7	−629.9	−560.6	N
	Obese (n = 1459)	−856.0	23.5	−902.0	−809.9	N
NHANES II	Normal (n = 3171)	−351.6	13.7	−378.5	−324.8	N
	Overweight (n = 1671)	−617.6	17.1	−651.1	−584.1	N
	Obese (n = 1164)	−850.6	19.5	−888.9	−812.3	N
NHANES III	Normal (n = 2661)	−158.6	17.7	−193.3	−123.9	N
	Overweight (n = 2150)	−357.1	26.5	−409.1	−305.2	N
	Obese (n = 2316)	−594.2	22.6	−638.5	−549.9	N
NHANES 1999–2000	Normal (n = 555)	−106.0	27.2	−159.3	−52.6	N
	Overweight (n = 572)	−359.6	48.8	−455.3	−264.0	N
	Obese (n = 759)	−530.1	50.2	−628.5	−431.6	N
NHANES 2001–2002	Normal (n = 630)	−74.0	21.7	−116.6	−31.4	N
	Overweight (n = 639)	−239.6	38.7	−315.5	−163.7	N
	Obese (n = 760)	−591.1	20.5	−631.4	−550.9	N
NHANES 2003–2004	Normal (n = 550)	−116.3	39.2	−193.2	−39.4	N
	Overweight (n = 546)	−339.0	37.7	−413.0	−265.0	N
	Obese (n = 742)	−477.1	42.2	−560.0	−394.2	N
NHANES 2005–2006	Normal (n = 615)	−131.1	34.1	−198.0	−64.3	N
	Overweight (n = 558)	−342.8	38.0	−417.4	−268.3	N
	Obese (n = 825)	−567.3	38.7	−643.2	−491.3	N
NHANES 2007–2008	Normal (n = 634)	−173.2	52.1	−275.4	−71.0	N
	Overweight (n = 694)	−374.1	35.8	−444.4	−303.7	N
	Obese (n = 978)	−567.3	33.2	−632.5	−502.1	N
NHANES 2009–2010	Normal (n = 690)	−173.0	27.8	−227.5	−118.4	N
	Overweight (n = 745)	−288.9	34.0	−355.7	−222.2	N
	Obese (n = 1097)	−590.5	14.0	−617.8	−563.1	N

Note: BMI = body mass index; TEE = estimated total energy expenditure; IOM = Institute of Medicine; rEI = reported energy intake; BMR = Basal Metabolic Rate calculated via Schofield predictive equation.

These values were calculated by subtracting the IOM TEE from the NHANES rEI for each respondent. Negative values indicate the kcal/day value of underreporting.

**Table 6:** Disparity between rEI and the TEE for all men (20–74 years) by BMI categories.

Disparity between rEI and IOM TEE; US Men by BMI categories (20–74 years) NHANES I – NHANES 2009–2010						
NHANESSurvey Year	BMI Category	Estimate rEI minus TEE (mean)	Standard Error	95% Confidence Interval (CI)		Validity: 95% CI includes zero (Y = Yes, N = No)
				Lower	Upper	
NHANES I	Normal (n = 2115)	−96.3	26.8	−149.0	−43.6	N
	Overweight (n = 1945)	−374.7	30.8	−435.1	−314.2	N
	Obese (n = 592)	−702.1	49.7	−799.7	−604.5	N
NHANES II	Normal (n = 2431)	−178.7	15.9	−209.9	−147.6	N
	Overweight (n = 2111)	−367.6	27.0	−420.5	−314.6	N
	Obese (n = 694)	−716.5	37.3	−789.8	−643.3	N
NHANES III	Normal (n = 2275)	−8.8	31.1	−69.8	52.2	Y
	Overweight (n = 2482)	−191.5	27.9	−246.3	−136.7	N
	Obese (n = 1365)	−494.4	38.0	−569.0	−419.9	N
NHANES 1999–2000	Normal (n = 476 )	−87.2	34.8	−155.6	−18.8	N
	Overweight (n = 655)	−221.8	41.5	−303.3	−140.2	N
	Obese (n 469)	−590.9	76.8	−741.6	−440.2	N
NHANES 2001–2002	Normal (n = 493)	−64.1	63.1	−188.0	59.9	Y
	Overweight (n = 774)	−229.2	29.5	−287.1	−171.3	N
	Obese (n = 515)	−527.5	55.3	−636.1	−418.9	N
NHANES 2003–2004	Normal (n = 465)	−6.8	47.3	−99.6	86.0	Y
	Overweight (n = 659)	−175.4	46.9	−267.4	−83.4	N
	Obese (n = 547)	−549.8	72.0	−691.1	−408.5	N
NHANES 2005–2006	Normal (n = 413)	70.4	53.0	−33.7	174.5	Y
	Overweight (n = 735)	−222.4	39.7	−300.3	−144.4	N
	Obese (n = 601)	−464.2	32.1	−527.2	−401.2	N
NHANES 2007–2008	Normal (n = 539)	−117.9	64.8	−245.2	9.3	Y
	Overweight (n = 835)	−286.7	31.3	−348.1	−225.2	N
	Obese (n = 790)	−608.0	42.2	−690.8	−525.2	N
NHANES 2009–2010	Normal (n = 563)	−154.4	43.5	−239.8	−69.1	N
	Overweight (n = 872 )	−178.9	42.1	−261.5	−96.4	N
	Obese (n = 884)	−590.9	32.9	−655.4	−526.4	N

Note: BMI = body mass index; TEE = estimated total energy expenditure; IOM = Institute of Medicine; rEI = reported energy intake; BMR = Basal Metabolic Rate calculated via Schofield predictive equation.

These values were calculated by subtracting the estimated IOM TEE from the NHANES rEI for each respondent. Negative numbers indicate the kcal/day value of underreporting.

### Trends in Underreporting

After the removal of over-reporters, both protocols, that is rEI/BMR (*Figure*1) and the disparity between rEI and IOM TEE (*Table* 4) exhibited significant decreases in underreporting from NHANES II and NHANES III (p<0.001). There were significant negative linear trends for both men and women in changes in underreporting total caloric intake from NHANES I to NHANES 2009–2010 (rEI/BMR: p<0.001, and disparity: p = 0.028).

### Trends in Over-reporting

Across the study period, approximately 4.9% of men and 2.9% of women reported rEI/BMR values suggestive of over-reporting (i.e., rEI/BMR >2.4) with no significant trends. The greatest increase in the percentage of over-reporters between survey periods occurred from NHANES II to NHANES III, with men increasing from 4.1% to 6.4%, and women from 1.7% to 3.4% (both p<0.001). The greatest absolute percentage of over-reporters was in NHANES III, with 6.4% of men over-reporting and NHANES 2003–2004, with 3.9% of women over-reporting.

## Discussion

### Validity of NHANES EI Data

Our results suggest that across the 39-year history of U.S. nutrition surveillance research, rEI data on the majority of respondents (67.3% of women and 58.7% of men) were not physiologically plausible. The historical average rEI/BMR values for all men and women were 1.31 and 1.19 respectively (*Table 1*). These values are indicative of substantial underreporting. The expected average values for healthy, free living men and women are ∼1.55, with a range of >1.35 to <2.40 [23], [27]. In no survey did at least 50% of the respondents report plausible EI values (*Figure 1*). These data are consistent with previous research demonstrating that the misreporting of EI in nutrition surveys is widespread [9], [11], [28]–[34]. Goldberg et al. (1991) demonstrated that in 37 studies across 10 countries, >65% of the mean rEI/BMR values were below the study-specific plausibility cut-off [23]. In addition to the extensive underreporting in our sample, 4.9% of men and 2.9% of women reported rEI/BMR values suggestive of over-reporting (i.e., rEI/BMR >2.40).

### Disparity between NHANES rEI and IOM Derived TEE

Throughout the study period (i.e., 1971–2010) the disparity between rEI and TEE values were large and variable across BMI and sex categories suggesting substantial systematic biases in underreporting (*Tables* 4, 5, 6). The overall mean disparity values for men and women were −281 kcal/day and −365 kcal/day, respectively. The greatest mean disparity values were −717 kcal/day (25% of TDEE) and −856 kcal/day (41% of TEE) in OB men and women, respectively.

### Trends in the Validity and Inferences from NHANES rEI Data

As depicted in Tables 1 and 2, and Figure 1, there were large decreases in underreporting between NHANES II and NHANES III. This is clearly evidenced by the increase in rEI/BMR index (*Table* 1), the large and significant increase in the percent of plausible reporters (*Figure*1), and the reduction in the disparity between NHANES rEI and NAS/IOM EER (*Table* 4). This decrement in underreporting between NHANES II and subsequent surveys across all sex and BMI categories is likely the result of improvements in survey protocols for NHANES III, such as the inclusion of more days of dietary recall (i.e., weekends), automated multi-pass methodology, and increased staff training and quality control (see [35]), The extent of these improvements is notable; for example, the percentage of OB women reporting implausible values decreased from ∼88% in NHANES II to 74% in NHANES III.

These changes in measurement protocols led to an apparent increase in mean rEI values that has been reported as an actual increase in population-level EI despite caveats that the “*Interpretation of trends in energy and nutrient intakes is difficult when methodologic changes occur between surveys*” [36]. Nevertheless, Briefel and Johnson state (without caveat) in their abstract, “*During the 30-year period, mean energy intake increased among adults…”* [37]. The data presented in the present report refute this inference. When the NHANES dietary measurement protocols were altered after NHANES II, the improved method captured a higher percentage of actual intakes. The apparent increase in mean rEI was merely an artifact of improved measurement protocols and not indicative of a true increase in caloric consumption. Despite this fact, the apparent increase has been regularly published and uncritically accepted as a true upward trend in caloric consumption (e.g., [37], [38]) and the cause of the obesity epidemic (e.g., [39], [40]).

### Changes in Underreporting and Public Policy Recommendations

In addition to the ubiquity of misreporting, there is strong evidence that the reporting of ‘socially undesirable’ (e.g., high fat and/or high sugar) foods has changed as the prevalence of obesity has increased [12]–[15]. Additionally, research has demonstrated that interventions emphasizing the importance of ‘healthy’ behaviors may lead to increased misreporting as participants alter their reports to reflect the adoption of the ‘healthier’ behaviors independent of actual behavior change [17], [41]. It appears that lifestyle interventions “teach” participants the socially desirable or acceptable responses [17], [42]. As such, the ubiquity of public health messages to ‘eat less and exercise more’ may induce greater levels of misreporting and may explain the recent downward bias in both self-reported EI [20] and body weight [17], [43], especially given that social desirability bias is often expressed in the underreporting of calorically dense foods [44].

Selective misreporting of specific macronutrients has important ramifications for epidemiological research and nutrition surveillance. Heitmann and Lissner (2005) demonstrated that the selective misreporting of dietary fat by groups at an increased risk of chronic non-communicable diseases may result in an overestimated association between fat consumption and disease [45]. If the potentially negative effects of high-fat diets are overestimated due to selective misreporting, current recommendations for fat intake may be overly conservative [45].

### Additional Systematic Biases of Nutrition Surveillance Data

In addition to known sources of systematic reporting error, there are numerous sources of systematic bias in nutrition surveillance research protocols that are not addressed via our data. Another potentially large source of error is the translation of food and beverage consumption data (e.g. 24HR) into nutrient energy values via nutrient composition databases. The accuracy of this translation relies on a number of assumptions that are rarely justified. As cited earlier, research on misreporting shows that reports do not accurately reflect the quantity or number of foods consumed, and are not representative of usual intakes [12]–[15], [46]–[50]. Given that the basic methodological assumptions are violated, it is not surprising that research has demonstrated that food data to nutrient energy conversions are “*riddled with potential pitfalls at all stages*” that “*hamper the interpretability of the results*” [51]–[53], and represent a major source of systematic error in national nutrition surveillance efforts [2].

Throughout its history, the NHANES has relied upon databases of varying quality and composition for the *post-hoc* conversion of food and beverage consumption (i.e., 24HR) data into energy values [2]–[5], [53]. This makes the analysis of trends extremely complex because the nutrient energy (i.e., caloric) values in the databases varied considerably over time [54], [55]. Additionally, research has demonstrated that the energy content of restaurant food (and especially fast-food outlets) vary significantly when compared to the industry values used in the NNDS [56], and an internal quality review of NHANES 2003–2004 data led to ∼400 substantive changes in nutrient and energy values. [57]. The result of these limitations are discussed in detail elsewhere, see [4], [5], [58].

As with the improvements in the NHANES survey protocols, the progressive alterations to the nutrient database combined with changes in the types of foods that are available for consumption led to artifactual differences in nutrient and energy consumption estimates that frustrate efforts to examine trends in caloric consumption [58]. To account for these changes, researchers must maintain the real differences in the composition of foods while correcting for artifactual differences attributable to improvements in the quality of nutrient data [58]. Given the lack of comprehensive crossover studies and metrics for adjustment as the food and nutrient databases evolved, papers examining trends in caloric consumption must be treated with skepticism [51], [58].

### Commercially Prepared Foods and Meals Away From Home

One of the most prominent systematic errors from 24HR data-to-nutrient energy conversions is due to the increased reliance on the food service industry and the substantial rise in meals eaten ‘away from home’[59]–[61]. As stated previously, the vast majority of foods and beverages in the NNDS have not been evaluated empirically and research has demonstrated that the energy and macro/micro nutrient content of commercially prepared foods varies significantly compared to the industry values used in the NNDS [56]. When foods or commodities are not in the database, substitutions are necessitated. For these interpolations to be accurate, the analogues must be similar in composition to the consumed food or beverage. This is extremely difficult to perform in practice because no two foods or commodities are identical, and local vs. imported foods/commodities differ significantly. For example, in survey data collection, knowledge of the specific preparation and cut of beef are essential since the energy content of generic beef substitutions may differ dramatically (e.g., 166 kcals per 100 grams in round steak to 257 kcals in top sirloin [62]) [63], [64]. Given these realities, USDA estimates of caloric consumption may be increasingly inaccurate as the number of food and beverages supplied by the commercial sector expands rapidly.

Recent research has attempted to quantify the changes in consumer packaged foods and beverages, and their impact on the American diet [65]. Nevertheless, these efforts suffer from the same limitations as all food data-to-nutrient energy value conversions via nutrient composition databases. Additionally, the translation of “as-purchased” foods and beverages (using information from the commercial sector) to “as-consumed” energy and macro/micronutrient content for national surveillance relies on the accurate quantification of food preparation and waste [65]. Unfortunately, these data are limited and highly variable [52], [66]. In a report from the USDA’s Economic Research Service, Muth et al. (2011) state that the current data are incomplete and overstate actual consumption because the level of “*documentation of food losses… ranged from little to none for estimates at the retail and customer levels*.” [67]. These results clearly demonstrate the conceptual and methodological complexity of translating food and beverage purchases into nutrient energy and macro/micronutrient intake in the context of a rapidly evolving food supply.

### Methods of Adjustment for Systematic Biases

There are various methods that attempt to improve estimates of caloric consumption derived from self-reported dietary intake [32], [68]–[72]. While these methods may improve the shape of the distribution of the estimates, none can address the significant systematic biases described in this report. For example, the National Research Council and the Iowa State University methods provide significantly improved estimates of the shape of the distribution, but do not substantially improve estimates of mean energy intake (10–15% underestimation) or protein consumption (6–7% underestimation) [70]. 291.

### Strengths and Limitations

A strength of the present study was the use of the established rEI/BMR method for the determination of physiologically implausible EI values. We used a liberal cutoff (i.e., <1.35) that is below the study-specific theoretical cutoff for our smallest sub-group (i.e., *n* >400). The use of the more conservative cutoff of rEI/BMR <1.50 recommended by Goldberg et al., (1991) [22] increased underreporting by 10% in women and 7% in men across all surveys. A second strength was the use of a rEI/BMR >2.4 for the elimination of potential over-reporters to correct the limitations of previous research [29].

Finally, the use of the IOM factorial equations for estimating TEE for specific subgroups (i.e., OW & OB respondents) in the calculation of disparity values is a significant strength. The results of this additional protocol demonstrated significant underreporting in all surveys, and that the disparity values closely paralleled the implausible values in 15 of the 18 sub-groups (i.e., men & women in 9 surveys). The close agreement between these two dissimilar protocols increases confidence in our results and conclusions.

A potential limitation to our analysis was the use of the Schofield predictive equation for estimating BMR. The Schofield predictive equations may overestimate BMR in some populations [73], [74]. If the Schofield equation overestimated BMR, a greater percentage of survey respondents would be classified as under-reporters. To address this potential limitation, we performed the analyses using the Mifflin equation [75], which has been validated in OW and OB populations such as the U.S [74]. The results of those analyses were similar to those obtained using the Schofield equation, with substantial underreporting (>50%) in all surveys, significant trends in changes in underreporting, and a small increase in over-reporting. To remain consistent with past research on implausible rEI and underreporting [29], [33], we chose to present the results from the Schofield predictive equations.

## Conclusions

Throughout its history, NHANES dietary measurement protocols have failed to provide accurate estimates of the habitual caloric consumption of the U.S. population. Furthermore, successive changes to the nutrient databases used for the 24HR data-to-energy conversations and improvements in measurement protocols make it exceedingly difficult to discern temporal patterns in caloric intake that can be related to changes in population rates of obesity. As such, there are no valid population-level data to support speculations regarding trends in caloric consumption and the etiology of the obesity epidemic. Because under-reporting and physiologically implausible rEI values are a predominant feature of U.S. nutritional surveillance, the ability to generate empirically supported public policy and dietary guidelines relevant to the obesity epidemic based on these data is extremely limited.
