# Correction: Validity of U.S. Nutritional Surveillance: National Health and Nutrition Examination Survey Caloric Energy Intake Data, 1971–2010

**DOI:** 10.1371/annotation/c313df3a-52bd-4cbe-af14-6676480d1a43

**Published:** 2013-10-11

**Authors:** Edward Archer, Gregory A. Hand, Steven N. Blair

The was an error in the Competing Interests section. The correct version of the section is available below.

Dr. Gregory Hand has received consultancy fees from the National Institutes of Health (NIH) and grants from the NIH, and The Coca-Cola Company. Dr. Steven Blair receives book royalties (<$5,000/year) from Human Kinetics; honoraria for service on the Scientific/Medical Advisory Boards for Clarity, Technogym, Santech, and Jenny Craig; and honoraria for lectures and consultations from scientific, educational, and lay groups which are donated to the University of South Carolina or not-for-profit organizations. Dr. Blair is a consultant on research projects with the University of Texas-Southwestern Medical School and the University of Miami. During the past 5-year period Dr. Blair has received research grants from The Coca-Cola Company, the National Institutes of Health, and Department of Defense. Funding for the study was provided by an unrestricted research grant from The Coca-Cola Company. The sponsor of the study had no role in the study design, data collection, data analysis, data interpretation, or writing of the report, and does not alter the authors' adherence to all the PLOS ONE policies on sharing data and materials.

The corresponding author should be contacted at: archer.edwardc@gmail.com

